# 1-{2-[(4-Chloro­benzyl­idene)amino]phen­yl}-3-phenyl­thio­urea

**DOI:** 10.1107/S1600536812006228

**Published:** 2012-02-17

**Authors:** Peng-Gang Liu, Xiao-Ning Wang, Yong-An Yang, Hai-Liang Zhu

**Affiliations:** a State Key Laboratory of Pharmaceutical Biotechnology, Nanjing University, Nanjing 210093, People’s Republic of China

## Abstract

The asymmetric unit of the title compound, C_20_H_16_ClN_3_S, contains two independent mol­ecules, *A* and *B*. In mol­ecule *A*, the dihedral angles between the central benzene ring and the pendant chloro­benzene and phenyl rings are 6.37 (15) and 64.79 (15)°, respectively. The corresponding values in mol­ecule *B* are 28.21 (14) and 82.11 (16)°, respectively. Each mol­ecule features an intra­molecular N—H⋯N hydrogen bond, which generates an *S*(5) ring. In the crystal, mol­ecules *A* and *B* form dimers, being linked by two N—H⋯S hydrogen bonds with graph-set notation *R*_2_^2^(8).

## Related literature

For background to the coordination chemistry of Schiff bases, see: Chen *et al.* (2010 ▶); Cui *et al.* (2011 ▶). For hydrogen-bond motifs, see: Bernstein *et al.* (1995 ▶).
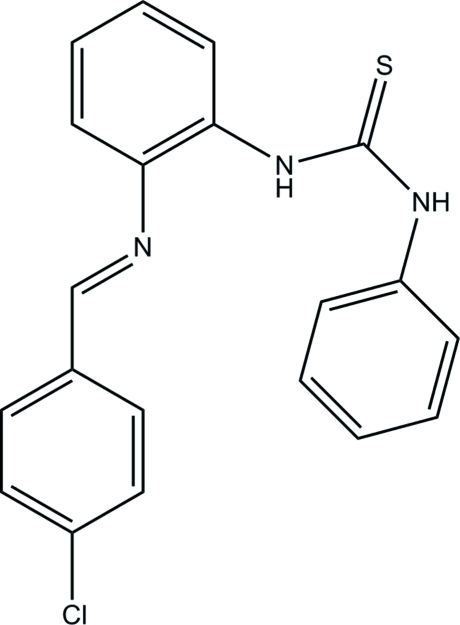


## Experimental

### 

#### Crystal data

C_20_H_16_ClN_3_S
*M* *_r_* = 365.87
Monoclinic, 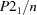

*a* = 9.410 (3) Å
*b* = 23.079 (3) Å
*c* = 16.807 (2) Å
β = 100.226 (2)°
*V* = 3592.0 (13) Å^3^
*Z* = 8
Mo *K*α radiation
μ = 0.34 mm^−1^
*T* = 298 K
0.17 × 0.15 × 0.15 mm

#### Data collection

Bruker SMART CCD area-detector diffractometer
Absorption correction: multi-scan (*SADABS*; Sheldrick, 1996 ▶) *T*_min_ = 0.945, *T*_max_ = 0.951
25609 measured reflections
6680 independent reflections
4051 reflections with *I* > 2σ(*I*)
*R*_int_ = 0.064

#### Refinement

*R*[*F* ^2^ > 2σ(*F* ^2^)] = 0.049
*wR*(*F* ^2^) = 0.117
*S* = 1.00
6680 reflections
463 parameters
4 restraints
H atoms treated by a mixture of independent and constrained refinement
Δρ_max_ = 0.31 e Å^−3^
Δρ_min_ = −0.27 e Å^−3^

### 

Data collection: *SMART* (Bruker, 1998 ▶); cell refinement: *SAINT* (Bruker, 1998 ▶); data reduction: *SAINT*; program(s) used to solve structure: *SHELXS97* (Sheldrick, 2008 ▶); program(s) used to refine structure: *SHELXL97* (Sheldrick, 2008 ▶); molecular graphics: *SHELXTL* (Sheldrick, 2008 ▶); software used to prepare material for publication: *SHELXTL*.

## Supplementary Material

Crystal structure: contains datablock(s) global, I. DOI: 10.1107/S1600536812006228/hb6632sup1.cif

Structure factors: contains datablock(s) I. DOI: 10.1107/S1600536812006228/hb6632Isup2.hkl

Supplementary material file. DOI: 10.1107/S1600536812006228/hb6632Isup3.cml

Additional supplementary materials:  crystallographic information; 3D view; checkCIF report

## Figures and Tables

**Table 1:** Hydrogen-bond geometry (Å, °)

*D*—H⋯*A*	*D*—H	H⋯*A*	*D*⋯*A*	*D*—H⋯*A*
N6—H6⋯S1^i^	0.89 (1)	2.77 (1)	3.647 (3)	168 (3)
N2—H2⋯N1	0.90 (1)	2.06 (3)	2.598 (3)	118 (3)
N5—H5⋯N4	0.90 (1)	2.16 (3)	2.637 (3)	113 (2)
N3—H3⋯S2^i^	0.91 (1)	2.42 (2)	3.282 (2)	160 (3)

Symmetry code: (i) 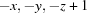.

**Fig. 1.**
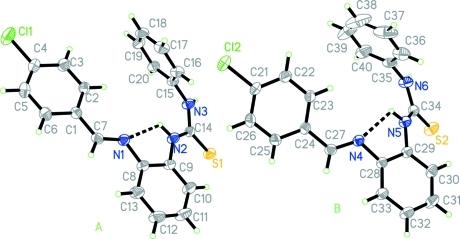
The molecular structure of the title compound, showing displacement ellipsoids drawn at the 30% probability level. Intramolecular hydrogen bonds are indicated by dashed lines.

**Fig. 2.**
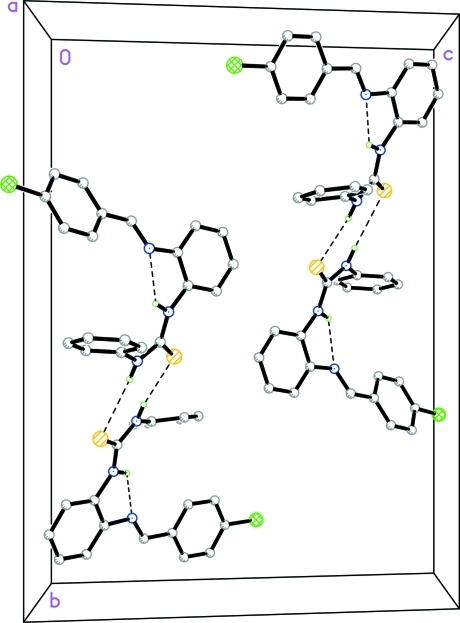
The molecular packing of the title compound, viewed along the a axis. Hydrogen bonds are indicated by dashed lines.

## supplementary crystallographic information

### Comment

Schiff bases are a kind of versatile ligands in the preparation of complexes (e.g. Chen *et al.*, 2010; Cui *et al.*, 2011). In the present paper, the title compound is reported.

There are two independent molecules, A and B, in the asymmetric unit of the title compound (Fig. 1). In A, the C8–C13 benzene ring forms dihedral angles of 6.1 (3) and 65.1 (3)° with the C1–C6 and C15–C20 benzene rings, respectively. The dihedral angle between the C1–C6 and C15–C20 benzene rings is 68.9 (3)°. In B, the C28–C33 benzene ring forms dihedral angles of 27.5 (3) and 82.8 (3)° with the C21–C26 and C35–C40 benzene rings, respectively. The dihedral angle between the C21–C26 and C35–C40 benzene rings is 70.6 (3)°. There forms an intramolecular N—H···N hydrogen bond in each molecule (Table 1).

### Experimental

*o*-Diaminobenzene from the plant Sophora Alopecuroides (1.85 mmol) was dissolved in ethanol (4 ml) and stirred at 293 K, a solution of phenyl isothiocyanate (0.22 ml) and ethanol was dropwised into the reaction mixture when the diaminobenzene was completely dissolved. The reaction mixture was stirred until the solids formed largely. The products were filtrated and washed carefully with EtOH; the resulting *N*-(2-amino)phenyl-*N*'-phenyl-thiourea were purified by crystallization from EtOH in refrigerator. *N*-(2-Amino)phenyl-*N*'-phenyl-thiourea (2.5 mmol) was dissolved in ethanol (10 ml) and stirred 353 K, salicylaldehyde (3.0 mmol) was dropwised into the solution when the solid was completely dissolved. The reaction mixture was stirred for 2.5 h. The product was filtrated timely and dried by vacuum. Yellow block-shaped single crystals were obtained by slow evaporation of the methanolic solution containing the compound in air.

### Refinement

The amino H atoms were located from a difference Fourier map and refined isotropically, with the N—H distances restrained to 0.90 (1) Å, and with *U*_iso_ restrained to 0.08 Å^2^. Other H atoms were constrained to ideal geometries, with C—H = 0.93 Å, and with *U*_iso_(H) = 1.2*U*_eq_(C).

### Crystal data

**Table:**

C_20_H_16_ClN_3_S	*F*(000) = 1520
*M**_r_* = 365.87	*D*_x_ = 1.353 Mg m^−^^3^
Monoclinic, *P*2_1_/*n*	Mo *K*α radiation, λ = 0.71073 Å
*a* = 9.410 (3) Å	Cell parameters from 4472 reflections
*b* = 23.079 (3) Å	θ = 2.4–24.5°
*c* = 16.807 (2) Å	µ = 0.34 mm^−^^1^
β = 100.226 (2)°	*T* = 298 K
*V* = 3592.0 (13) Å^3^	Block, yellow
*Z* = 8	0.17 × 0.15 × 0.15 mm

### Data collection

**Table:**

Bruker SMART CCD area-detector diffractometer	6680 independent reflections
Radiation source: fine-focus sealed tube	4051 reflections with *I* > 2σ(*I*)
Graphite monochromator	*R*_int_ = 0.064
ω scans	θ_max_ = 25.5°, θ_min_ = 2.2°
Absorption correction: multi-scan (*SADABS*; Sheldrick, 1996)	*h* = −11→11
*T*_min_ = 0.945, *T*_max_ = 0.951	*k* = −27→26
25609 measured reflections	*l* = −20→20

### Refinement

**Table:**

Refinement on *F*^2^	Primary atom site location: structure-invariant direct methods
Least-squares matrix: full	Secondary atom site location: difference Fourier map
*R*[*F*^2^ > 2σ(*F*^2^)] = 0.049	Hydrogen site location: inferred from neighbouring sites
*wR*(*F*^2^) = 0.117	H atoms treated by a mixture of independent and constrained refinement
*S* = 1.00	*w* = 1/[σ^2^(*F*_o_^2^) + (0.0334*P*)^2^ + 1.8733*P*] where *P* = (*F*_o_^2^ + 2*F*_c_^2^)/3
6680 reflections	(Δ/σ)_max_ < 0.001
463 parameters	Δρ_max_ = 0.31 e Å^−^^3^
4 restraints	Δρ_min_ = −0.27 e Å^−^^3^

### Special details

**Table:**

Geometry. All e.s.d.'s (except the e.s.d. in the dihedral angle between two l.s. planes) are estimated using the full covariance matrix. The cell e.s.d.'s are taken into account individually in the estimation of e.s.d.'s in distances, angles and torsion angles; correlations between e.s.d.'s in cell parameters are only used when they are defined by crystal symmetry. An approximate (isotropic) treatment of cell e.s.d.'s is used for estimating e.s.d.'s involving l.s. planes.
Refinement. Refinement of *F*^2^ against ALL reflections. The weighted *R*-factor *wR* and goodness of fit *S* are based on *F*^2^, conventional *R*-factors *R* are based on *F*, with *F* set to zero for negative *F*^2^. The threshold expression of *F*^2^ > σ(*F*^2^) is used only for calculating *R*-factors(gt) *etc*. and is not relevant to the choice of reflections for refinement. *R*-factors based on *F*^2^ are statistically about twice as large as those based on *F*, and *R*-factors based on ALL data will be even larger.

### Fractional atomic coordinates and isotropic or equivalent isotropic displacement parameters (Å^2^)

**Table:**

	*x*	*y*	*z*	*U*_iso_*/*U*_eq_	
Cl1	0.63112 (12)	0.39717 (5)	−0.02622 (6)	0.0897 (3)	
Cl2	0.63346 (9)	0.20059 (4)	0.47794 (5)	0.0672 (3)	
N1	0.2757 (2)	0.37248 (10)	0.28692 (14)	0.0453 (6)	
N2	0.1379 (3)	0.28279 (10)	0.32759 (15)	0.0494 (6)	
N3	0.1149 (3)	0.19376 (10)	0.27030 (15)	0.0496 (6)	
N4	0.2947 (2)	0.10566 (9)	0.77336 (13)	0.0396 (6)	
N5	0.2405 (3)	−0.00077 (10)	0.81666 (14)	0.0462 (6)	
N6	0.2450 (3)	−0.08378 (10)	0.74386 (17)	0.0540 (7)	
S1	−0.09328 (8)	0.21834 (4)	0.34837 (5)	0.0560 (2)	
S2	0.03254 (10)	−0.07857 (4)	0.82505 (5)	0.0639 (3)	
C1	0.4126 (3)	0.40775 (12)	0.18976 (17)	0.0421 (7)	
C2	0.4179 (3)	0.35595 (13)	0.14910 (18)	0.0509 (8)	
H2A	0.3767	0.3230	0.1673	0.061*	
C3	0.4832 (3)	0.35242 (14)	0.08212 (19)	0.0573 (9)	
H3A	0.4857	0.3174	0.0549	0.069*	
C4	0.5446 (3)	0.40117 (15)	0.05601 (18)	0.0549 (8)	
C5	0.5406 (3)	0.45284 (14)	0.09521 (19)	0.0584 (9)	
H5A	0.5830	0.4856	0.0772	0.070*	
C6	0.4738 (3)	0.45629 (13)	0.16128 (18)	0.0532 (8)	
H6A	0.4697	0.4917	0.1872	0.064*	
C7	0.3428 (3)	0.41324 (12)	0.25979 (17)	0.0453 (7)	
H7	0.3477	0.4488	0.2862	0.054*	
C8	0.2020 (3)	0.38065 (13)	0.35255 (17)	0.0448 (7)	
C9	0.1256 (3)	0.33273 (12)	0.37331 (17)	0.0441 (7)	
C10	0.0506 (3)	0.33636 (14)	0.43690 (19)	0.0599 (9)	
H10	0.0021	0.3042	0.4519	0.072*	
C11	0.0481 (4)	0.38780 (16)	0.4776 (2)	0.0720 (11)	
H11	−0.0041	0.3904	0.5196	0.086*	
C12	0.1207 (4)	0.43513 (16)	0.4576 (2)	0.0707 (10)	
H12	0.1178	0.4697	0.4856	0.085*	
C13	0.1980 (3)	0.43156 (14)	0.3960 (2)	0.0620 (9)	
H13	0.2487	0.4638	0.3831	0.074*	
C14	0.0607 (3)	0.23353 (12)	0.31572 (16)	0.0430 (7)	
C15	0.2432 (3)	0.19785 (11)	0.23773 (18)	0.0420 (7)	
C16	0.3757 (3)	0.20611 (12)	0.2862 (2)	0.0533 (8)	
H16	0.3822	0.2106	0.3417	0.064*	
C17	0.4981 (3)	0.20764 (13)	0.2527 (2)	0.0611 (9)	
H17	0.5875	0.2136	0.2854	0.073*	
C18	0.4892 (4)	0.20044 (13)	0.1708 (2)	0.0623 (10)	
H18	0.5725	0.2014	0.1482	0.075*	
C19	0.3582 (4)	0.19191 (14)	0.1227 (2)	0.0619 (9)	
H19	0.3525	0.1867	0.0673	0.074*	
C20	0.2348 (3)	0.19091 (13)	0.15560 (18)	0.0519 (8)	
H20	0.1455	0.1856	0.1225	0.062*	
C21	0.5270 (3)	0.18491 (13)	0.54944 (16)	0.0462 (7)	
C22	0.5486 (3)	0.13397 (13)	0.59180 (17)	0.0492 (8)	
H22	0.6169	0.1074	0.5808	0.059*	
C23	0.4681 (3)	0.12249 (12)	0.65079 (16)	0.0438 (7)	
H23	0.4824	0.0880	0.6798	0.053*	
C24	0.3664 (3)	0.16162 (12)	0.66733 (16)	0.0410 (7)	
C25	0.3474 (3)	0.21226 (13)	0.62326 (19)	0.0581 (9)	
H25	0.2795	0.2391	0.6340	0.070*	
C26	0.4263 (3)	0.22396 (14)	0.56382 (19)	0.0578 (9)	
H26	0.4112	0.2580	0.5339	0.069*	
C27	0.2832 (3)	0.15137 (12)	0.73111 (16)	0.0435 (7)	
H27	0.2186	0.1797	0.7413	0.052*	
C28	0.2163 (3)	0.10037 (11)	0.83720 (16)	0.0371 (6)	
C29	0.1911 (3)	0.04411 (11)	0.86153 (16)	0.0393 (7)	
C30	0.1266 (3)	0.03522 (13)	0.92816 (17)	0.0515 (8)	
H30	0.1132	−0.0022	0.9458	0.062*	
C31	0.0822 (3)	0.08202 (14)	0.96840 (18)	0.0593 (9)	
H31	0.0378	0.0760	1.0129	0.071*	
C32	0.1031 (3)	0.13720 (14)	0.94326 (18)	0.0578 (9)	
H32	0.0704	0.1685	0.9699	0.069*	
C33	0.1717 (3)	0.14676 (12)	0.87925 (17)	0.0480 (8)	
H33	0.1885	0.1845	0.8638	0.058*	
C34	0.1802 (3)	−0.05245 (12)	0.79539 (17)	0.0440 (7)	
C35	0.3686 (4)	−0.06772 (12)	0.7116 (2)	0.0520 (8)	
C36	0.4987 (4)	−0.05871 (14)	0.7615 (3)	0.0716 (10)	
H36	0.5053	−0.0612	0.8173	0.086*	
C37	0.6190 (4)	−0.04602 (17)	0.7293 (4)	0.1041 (17)	
H37	0.7072	−0.0399	0.7630	0.125*	
C38	0.6087 (6)	−0.0425 (2)	0.6481 (4)	0.130 (2)	
H38	0.6904	−0.0338	0.6264	0.155*	
C39	0.4806 (7)	−0.0514 (2)	0.5980 (3)	0.121 (2)	
H39	0.4747	−0.0489	0.5423	0.145*	
C40	0.3587 (4)	−0.06431 (15)	0.6302 (2)	0.0791 (11)	
H40	0.2706	−0.0706	0.5963	0.095*	
H3	0.056 (3)	0.1640 (10)	0.2513 (18)	0.080*	
H5	0.306 (3)	0.0129 (13)	0.7888 (17)	0.080*	
H2	0.206 (3)	0.2887 (14)	0.2977 (16)	0.080*	
H6	0.196 (3)	−0.1152 (9)	0.7242 (18)	0.080*	

### Atomic displacement parameters (Å^2^)

**Table:**

	*U*^11^	*U*^22^	*U*^33^	*U*^12^	*U*^13^	*U*^23^
Cl1	0.1045 (8)	0.1059 (8)	0.0690 (6)	−0.0180 (6)	0.0435 (6)	0.0027 (6)
Cl2	0.0719 (6)	0.0827 (6)	0.0542 (5)	−0.0080 (5)	0.0309 (4)	0.0078 (4)
N1	0.0449 (15)	0.0425 (15)	0.0515 (15)	−0.0055 (12)	0.0168 (12)	−0.0050 (12)
N2	0.0490 (16)	0.0439 (15)	0.0614 (17)	−0.0128 (13)	0.0268 (13)	−0.0102 (13)
N3	0.0427 (16)	0.0472 (16)	0.0636 (17)	−0.0115 (12)	0.0223 (13)	−0.0136 (13)
N4	0.0411 (14)	0.0353 (13)	0.0456 (14)	−0.0071 (11)	0.0169 (11)	−0.0012 (11)
N5	0.0530 (16)	0.0330 (14)	0.0586 (16)	−0.0110 (12)	0.0263 (13)	−0.0028 (12)
N6	0.0528 (17)	0.0396 (15)	0.0752 (18)	−0.0098 (12)	0.0266 (15)	−0.0134 (13)
S1	0.0452 (5)	0.0555 (5)	0.0733 (6)	−0.0057 (4)	0.0265 (4)	−0.0005 (4)
S2	0.0714 (6)	0.0557 (5)	0.0735 (6)	−0.0298 (4)	0.0366 (5)	−0.0149 (4)
C1	0.0356 (17)	0.0412 (17)	0.0486 (18)	−0.0033 (13)	0.0048 (14)	0.0014 (14)
C2	0.0507 (19)	0.0415 (18)	0.064 (2)	−0.0044 (15)	0.0194 (16)	0.0037 (15)
C3	0.059 (2)	0.053 (2)	0.063 (2)	−0.0094 (16)	0.0191 (18)	−0.0069 (16)
C4	0.053 (2)	0.065 (2)	0.0474 (19)	−0.0069 (17)	0.0125 (16)	0.0057 (17)
C5	0.061 (2)	0.056 (2)	0.059 (2)	−0.0158 (17)	0.0102 (18)	0.0096 (17)
C6	0.055 (2)	0.0438 (19)	0.060 (2)	−0.0093 (15)	0.0083 (17)	−0.0033 (16)
C7	0.0435 (18)	0.0360 (17)	0.0564 (19)	−0.0006 (14)	0.0091 (15)	−0.0045 (14)
C8	0.0413 (18)	0.0467 (18)	0.0480 (18)	−0.0018 (14)	0.0120 (15)	−0.0066 (14)
C9	0.0405 (17)	0.0468 (18)	0.0468 (17)	−0.0014 (14)	0.0123 (14)	−0.0083 (14)
C10	0.063 (2)	0.062 (2)	0.062 (2)	−0.0146 (17)	0.0292 (18)	−0.0150 (17)
C11	0.072 (2)	0.084 (3)	0.068 (2)	−0.015 (2)	0.035 (2)	−0.031 (2)
C12	0.073 (3)	0.067 (2)	0.079 (3)	−0.008 (2)	0.031 (2)	−0.031 (2)
C13	0.063 (2)	0.053 (2)	0.075 (2)	−0.0098 (17)	0.0256 (19)	−0.0158 (18)
C14	0.0417 (18)	0.0441 (18)	0.0446 (17)	−0.0018 (14)	0.0115 (14)	0.0039 (14)
C15	0.0366 (17)	0.0357 (16)	0.0560 (19)	−0.0053 (13)	0.0143 (15)	−0.0076 (14)
C16	0.043 (2)	0.0516 (19)	0.065 (2)	−0.0057 (15)	0.0072 (17)	−0.0106 (16)
C17	0.0367 (19)	0.051 (2)	0.093 (3)	−0.0062 (15)	0.0049 (18)	−0.0107 (19)
C18	0.048 (2)	0.048 (2)	0.099 (3)	−0.0031 (16)	0.038 (2)	−0.0011 (19)
C19	0.066 (2)	0.063 (2)	0.064 (2)	0.0006 (19)	0.032 (2)	−0.0002 (18)
C20	0.0467 (19)	0.056 (2)	0.054 (2)	−0.0030 (16)	0.0122 (16)	−0.0037 (16)
C21	0.0452 (19)	0.057 (2)	0.0398 (17)	−0.0072 (15)	0.0155 (14)	0.0023 (15)
C22	0.0450 (19)	0.058 (2)	0.0459 (18)	0.0050 (15)	0.0110 (15)	0.0004 (15)
C23	0.0439 (18)	0.0454 (17)	0.0425 (17)	−0.0005 (14)	0.0085 (14)	0.0069 (14)
C24	0.0415 (17)	0.0419 (17)	0.0412 (16)	−0.0080 (14)	0.0120 (14)	0.0021 (13)
C25	0.063 (2)	0.0445 (18)	0.074 (2)	0.0083 (16)	0.0326 (18)	0.0138 (17)
C26	0.065 (2)	0.050 (2)	0.063 (2)	−0.0006 (17)	0.0235 (18)	0.0198 (16)
C27	0.0451 (18)	0.0389 (17)	0.0493 (18)	−0.0029 (14)	0.0161 (15)	−0.0033 (14)
C28	0.0367 (16)	0.0363 (15)	0.0403 (16)	−0.0071 (12)	0.0125 (13)	−0.0022 (13)
C29	0.0389 (17)	0.0374 (16)	0.0433 (16)	−0.0092 (13)	0.0118 (14)	−0.0025 (13)
C30	0.062 (2)	0.0483 (19)	0.0479 (18)	−0.0190 (16)	0.0195 (16)	−0.0002 (15)
C31	0.071 (2)	0.063 (2)	0.0509 (19)	−0.0208 (18)	0.0312 (18)	−0.0123 (17)
C32	0.068 (2)	0.054 (2)	0.057 (2)	−0.0108 (17)	0.0280 (18)	−0.0163 (16)
C33	0.058 (2)	0.0384 (17)	0.0521 (19)	−0.0102 (14)	0.0215 (16)	−0.0059 (14)
C34	0.0496 (19)	0.0340 (16)	0.0503 (18)	−0.0050 (14)	0.0138 (15)	0.0012 (14)
C35	0.053 (2)	0.0308 (16)	0.078 (2)	−0.0016 (15)	0.0276 (19)	−0.0131 (16)
C36	0.058 (2)	0.050 (2)	0.110 (3)	−0.0070 (18)	0.024 (2)	−0.012 (2)
C37	0.062 (3)	0.065 (3)	0.194 (5)	−0.013 (2)	0.048 (3)	−0.047 (3)
C38	0.127 (5)	0.089 (3)	0.206 (6)	−0.048 (3)	0.120 (5)	−0.074 (4)
C39	0.162 (5)	0.111 (4)	0.116 (4)	−0.053 (4)	0.096 (4)	−0.049 (3)
C40	0.091 (3)	0.072 (3)	0.083 (3)	−0.010 (2)	0.038 (2)	−0.021 (2)

### Geometric parameters (Å, º)

**Table:**

Cl1—C4	1.726 (3)	C15—C20	1.378 (4)
Cl2—C21	1.733 (3)	C16—C17	1.370 (4)
N1—C7	1.262 (3)	C16—H16	0.9300
N1—C8	1.416 (3)	C17—C18	1.374 (4)
N2—C14	1.345 (3)	C17—H17	0.9300
N2—C9	1.401 (3)	C18—C19	1.363 (4)
N2—H2	0.896 (10)	C18—H18	0.9300
N3—C14	1.350 (3)	C19—C20	1.373 (4)
N3—C15	1.415 (3)	C19—H19	0.9300
N3—H3	0.905 (10)	C20—H20	0.9300
N4—C27	1.266 (3)	C21—C26	1.360 (4)
N4—C28	1.411 (3)	C21—C22	1.371 (4)
N5—C34	1.342 (3)	C22—C23	1.376 (4)
N5—C29	1.408 (3)	C22—H22	0.9300
N5—H5	0.895 (10)	C23—C24	1.380 (4)
N6—C34	1.354 (3)	C23—H23	0.9300
N6—C35	1.416 (4)	C24—C25	1.378 (4)
N6—H6	0.892 (10)	C24—C27	1.454 (3)
S1—C14	1.675 (3)	C25—C26	1.374 (4)
S2—C34	1.670 (3)	C25—H25	0.9300
C1—C2	1.382 (4)	C26—H26	0.9300
C1—C6	1.384 (4)	C27—H27	0.9300
C1—C7	1.452 (4)	C28—C33	1.388 (4)
C2—C3	1.378 (4)	C28—C29	1.394 (3)
C2—H2A	0.9300	C29—C30	1.381 (3)
C3—C4	1.372 (4)	C30—C31	1.378 (4)
C3—H3A	0.9300	C30—H30	0.9300
C4—C5	1.366 (4)	C31—C32	1.367 (4)
C5—C6	1.372 (4)	C31—H31	0.9300
C5—H5A	0.9300	C32—C33	1.367 (4)
C6—H6A	0.9300	C32—H32	0.9300
C7—H7	0.9300	C33—H33	0.9300
C8—C13	1.388 (4)	C35—C40	1.357 (4)
C8—C9	1.397 (4)	C35—C36	1.371 (5)
C9—C10	1.384 (4)	C36—C37	1.370 (5)
C10—C11	1.373 (4)	C36—H36	0.9300
C10—H10	0.9300	C37—C38	1.352 (7)
C11—C12	1.363 (5)	C37—H37	0.9300
C11—H11	0.9300	C38—C39	1.359 (7)
C12—C13	1.369 (4)	C38—H38	0.9300
C12—H12	0.9300	C39—C40	1.385 (5)
C13—H13	0.9300	C39—H39	0.9300
C15—C16	1.376 (4)	C40—H40	0.9300
			
C7—N1—C8	121.8 (2)	C17—C18—H18	120.0
C14—N2—C9	133.0 (2)	C18—C19—C20	120.3 (3)
C14—N2—H2	118 (2)	C18—C19—H19	119.9
C9—N2—H2	109 (2)	C20—C19—H19	119.9
C14—N3—C15	127.4 (2)	C19—C20—C15	120.0 (3)
C14—N3—H3	117 (2)	C19—C20—H20	120.0
C15—N3—H3	115 (2)	C15—C20—H20	120.0
C27—N4—C28	119.7 (2)	C26—C21—C22	121.3 (3)
C34—N5—C29	129.2 (2)	C26—C21—Cl2	119.4 (2)
C34—N5—H5	118 (2)	C22—C21—Cl2	119.3 (2)
C29—N5—H5	110 (2)	C21—C22—C23	119.3 (3)
C34—N6—C35	126.8 (2)	C21—C22—H22	120.3
C34—N6—H6	114 (2)	C23—C22—H22	120.3
C35—N6—H6	119 (2)	C22—C23—C24	120.6 (3)
C2—C1—C6	118.4 (3)	C22—C23—H23	119.7
C2—C1—C7	122.5 (3)	C24—C23—H23	119.7
C6—C1—C7	119.1 (3)	C25—C24—C23	118.4 (3)
C3—C2—C1	121.1 (3)	C25—C24—C27	119.9 (3)
C3—C2—H2A	119.5	C23—C24—C27	121.7 (3)
C1—C2—H2A	119.5	C26—C25—C24	121.5 (3)
C4—C3—C2	119.2 (3)	C26—C25—H25	119.3
C4—C3—H3A	120.4	C24—C25—H25	119.3
C2—C3—H3A	120.4	C21—C26—C25	118.9 (3)
C5—C4—C3	120.8 (3)	C21—C26—H26	120.6
C5—C4—Cl1	119.4 (2)	C25—C26—H26	120.6
C3—C4—Cl1	119.8 (3)	N4—C27—C24	123.1 (3)
C4—C5—C6	119.8 (3)	N4—C27—H27	118.5
C4—C5—H5A	120.1	C24—C27—H27	118.5
C6—C5—H5A	120.1	C33—C28—C29	119.1 (2)
C5—C6—C1	120.8 (3)	C33—C28—N4	124.5 (2)
C5—C6—H6A	119.6	C29—C28—N4	116.3 (2)
C1—C6—H6A	119.6	C30—C29—C28	119.9 (3)
N1—C7—C1	123.7 (3)	C30—C29—N5	124.0 (2)
N1—C7—H7	118.1	C28—C29—N5	116.0 (2)
C1—C7—H7	118.1	C31—C30—C29	119.8 (3)
C13—C8—C9	118.4 (3)	C31—C30—H30	120.1
C13—C8—N1	125.9 (3)	C29—C30—H30	120.1
C9—C8—N1	115.7 (2)	C32—C31—C30	120.4 (3)
C10—C9—C8	120.0 (3)	C32—C31—H31	119.8
C10—C9—N2	125.2 (3)	C30—C31—H31	119.8
C8—C9—N2	114.7 (2)	C31—C32—C33	120.5 (3)
C11—C10—C9	119.6 (3)	C31—C32—H32	119.8
C11—C10—H10	120.2	C33—C32—H32	119.8
C9—C10—H10	120.2	C32—C33—C28	120.3 (3)
C12—C11—C10	121.0 (3)	C32—C33—H33	119.9
C12—C11—H11	119.5	C28—C33—H33	119.9
C10—C11—H11	119.5	N5—C34—N6	115.3 (2)
C11—C12—C13	119.8 (3)	N5—C34—S2	125.2 (2)
C11—C12—H12	120.1	N6—C34—S2	119.5 (2)
C13—C12—H12	120.1	C40—C35—C36	120.1 (3)
C12—C13—C8	121.1 (3)	C40—C35—N6	119.1 (3)
C12—C13—H13	119.4	C36—C35—N6	120.7 (3)
C8—C13—H13	119.4	C37—C36—C35	120.1 (4)
N2—C14—N3	114.2 (2)	C37—C36—H36	120.0
N2—C14—S1	127.2 (2)	C35—C36—H36	120.0
N3—C14—S1	118.6 (2)	C38—C37—C36	119.7 (5)
C16—C15—C20	119.6 (3)	C38—C37—H37	120.1
C16—C15—N3	121.7 (3)	C36—C37—H37	120.1
C20—C15—N3	118.6 (3)	C37—C38—C39	120.8 (4)
C17—C16—C15	119.9 (3)	C37—C38—H38	119.6
C17—C16—H16	120.0	C39—C38—H38	119.6
C15—C16—H16	120.0	C38—C39—C40	119.7 (5)
C16—C17—C18	120.2 (3)	C38—C39—H39	120.1
C16—C17—H17	119.9	C40—C39—H39	120.1
C18—C17—H17	119.9	C35—C40—C39	119.5 (4)
C19—C18—C17	120.0 (3)	C35—C40—H40	120.2
C19—C18—H18	120.0	C39—C40—H40	120.2

### Hydrogen-bond geometry (Å, º)

**Table:**

*D*—H···*A*	*D*—H	H···*A*	*D*···*A*	*D*—H···*A*
N6—H6···S1^i^	0.89 (1)	2.77 (1)	3.647 (3)	168 (3)
N2—H2···N1	0.90 (1)	2.06 (3)	2.598 (3)	118 (3)
N5—H5···N4	0.90 (1)	2.16 (3)	2.637 (3)	113 (2)
N3—H3···S2^i^	0.91 (1)	2.42 (2)	3.282 (2)	160 (3)

Symmetry code: (i) −*x*, −*y*, −*z*+1.

## References

[bb1] Bernstein, J., Davis, R. E., Shimoni, L. & Chang, N.-L. (1995). *Angew. Chem. Int. Ed. Engl.* **34**, 1555–1573.

[bb2] Bruker (1998). *SMART* and *SAINT* Bruker AXS Inc., Madison, Wisconsin, USA.

[bb3] Chen, W., Li, Y.-G., Cui, Y.-M., Zhang, X.-A., Zhu, H.-L. & Zeng, Q.-F. (2010). *Eur. J. Med. Chem.* **45**, 4473–4478. 10.1016/j.ejmech.2010.07.007 20691510

[bb4] Cui, Y.-M., Li, Y.-G., Cai, Y.-J., Chen, W. & Zhu, H.-L. (2011). *J. Coord. Chem.* **64**, 610–616.

[bb5] Sheldrick, G. M. (1996). *SADABS* University of Göttingen, Germany.

[bb6] Sheldrick, G. M. (2008). *Acta Cryst.* A**64**, 112–122. 10.1107/S0108767307043930 18156677

